# Supplementary material for: Immune Response following BNT162b2 mRNA COVID-19 Vaccination in Pediatric Cancer Patients
Source: Cancers (Basel). 2023 Apr 29;15(9):2562. doi: 10.3390/cancers15092562 (PMC10177402; doi:10.3390/cancers15092562)
Supplement: Supplementary file 1 [file cancers-15-02562-s001.zip › cancers-2332169-supplementary.pdf]

cancers

MDPI

# Supplemental Materials: Immune Response following BNT162b2 mRNA COVID-19 Vaccination in Pediatric Cancer Patients

## Supplementary Table S1:

**Table S1. Baseline demographics and patient characteristics of patients without a history of COVID-19 receiving 2-dose vaccination (n = 51)**

| | **Tx < 6 weeks[a]** **n = 28** | **Tx > 6 weeks[a]** **n = 19** | **No Tx[a]** n = 4 |
|---|---|---|---|
| **Per age category, n (%)** | | | |
| 5-11 years | 11 (39.3) | 3 (15.8) | 0 |
| 12-17 years | 17 (60.7) | 16 (84.2) | 4 (100) |
| **Age, years (median, range)** | 12.5 (6-17) | 14 (6-17) | 13.5 (12-15) |
| **Sex, n (%)** | | | |
| Male | 14 (50) | 10 (52.6) | 3 (75) |
| Female | 14 (50) | 9 (47.4) | 1 (25) |
| **Diagnosis, n (%)** | | | |
| Acute lymphoblastic leukemia | 15 (53.6) | 6 (31.6) | 0 |
| Other hematological malignancies | 4 (14.3) | 9 (47.4) | 4 (100) |
| Solid tumors | 5 (17.9) | 4 (21.1) | 0 |
| CNS tumors | 3 (10.7) | 0 | 0 |
| Non malignancies[b] | 1 (3.6) | 0 | 0 |
| **History of chemotherapy, n (%)** | 28 (100) | 19 (100) | 0 |
| **History of immunotherapy, n (%)** | 1 (3.6) | 4 (21.1) | |
| Bevacizumab | 1 | 0 | |
| Blinatumomab | 0 | 1 | 0 |
| Rituximab | 0 | 2 | |
| Tocilizumab | 0 | 1 | |
| **History of recent IVIG[c], n (%)** | 1 (3.6) | 1 (5.3) | |
| **History of HSCT/CAR-T, n (%)** | 2 (7.1) | 7 (36.8) | |
| Allo-SCT | 2 (100) | 2 (28.6) | |
| Auto-SCT | 0 | 1 (14.3) | 0 |
| CAR T | 0 | 2 (28.6) | |
| CAR T + allo-SCT | 0 | 2 (28.6) | |
| **Antibody response - number of patients with data available** | | | |
| 28 days after the 2nd vaccination | 28 | 18 | 4 |
| 28 days after the 3rd vaccination | 10 | 6 | 0 |
| **T-cell response - number of patients with data available** | | | |
| 28 days after the 2nd vaccination | 19 | 12 | 3 |
| 28 days after the 3rd vaccination | 3 | 4 | 0 |

[a] Defined as receiving chemotherapy or immunotherapy less (Tx < 6 weeks) or more (Tx > 6 weeks) than 6 weeks before 1st vaccination or having no history of chemotherapy or immunotherapy (No Tx)
[b] Non malignancies: familial autosomal dominant primary immunodeficiency (RAC 2 mutation)
[c] IVIG (intravenous immunoglobulin) <2 months prior to 1st vaccination.

**Supplementary Table S2:**

**Table S2. Baseline demographics and patient characteristics of patients with a history of COVID-19 prior to or fol-lowing vaccination (hybrid immunity)**

| | **1 vaccination + SARS-CoV-2 infection[a]** | | | **2-dose vaccination + SARS-CoV-2 infection[b]** | | |
|---|---|---|---|---|---|---|
| | **Tx < 6 weeks[c]** | **Tx > 6 weeks[c]** | **No Tx[c]** | **Tx < 6 weeks[c]** | **Tx > 6 weeks[c]** | **No Tx[c]** |
| | **n = 9** | **n = 4** | **n = 1** | n = 4 | n = 7 | n = 1 |
| **Per age category, n (%)** | | | | | | |
| 5-11 years | 8 (88.9) | 2 (50) | 0 | 2 (50) | 2 (28.6) | 0 |
| 12-17 years | 1 (11.1) | 2 (50) | 1 (100) | 2 (50) | 5 (71.4) | 1 (100) |
| **Age, years (median, range)** | 8 (5-15) | 12.5 (7-17) | 16 | 11 (6-16) | 15 (5-16) | 16 |
| **Sex, n (%)** | | | | | | |
| Male | 6 (66.7) | 2 (50) | 0 | 3 (75) | 2 (28.6) | 0 |
| Female | 3 (33.3) | 2 (50) | 1 | 1 (25) | 5 (71.4) | 1 |
| **Diagnosis, n (%)** | | | | | | |
| Acute lymphoblastic leukemia | 4 (44.4) | 1 (25) | 0 | 2 (50) | 1 (14.3) | 0 |
| Other hematological malignancies | 2 (22.2) | 1 (25) | 0 | 1 (25) | 2 (28.6) | 0 |
| Solid tumors | 2 (22.2) | 1 (25) | 1 | 0 | 2 (28.6) | 1 |
| CNS tumors | 1 (11.1) | 0 | 0 | 1 (25) | 0 | 0 |
| Non malignancies[d] | 0 | 1 (25) | 0 | 0 | 2 (28.6) | 0 |
| **History of chemotherapy, n (%)** | 9 (100) | 4 (100) | 0 | 4 (100) | 7 (100) | 0 |
| **History of immunotherapy, n (%)** | 3 (33.3) | 2 (50) | 0 | 2 (50) | 1 (14.3) | 0 |
| Bevacizumab | 1 | 0 | | 1 | 0 | |
| Blinatumomab | 0 | 0 | | 0 | 1 | |
| Daratumumab | 0 | 1 | | 0 | 0 | |
| Dinutuximab | 1 | 0 | | 0 | 0 | |
| Basiliximab, infliximab, vedolizumab | 0 | 1 | | 0 | 0 | |
| Rituximab and Tocilizumab | 1 | 0 | | 1 | 0 | |
| **History of recent IVIG[e], n (%)** | 0 | 1 (33.3) | 0 | 0 | 1 (25) | 0 |
| **History of HSCT[f], n (%)** | 1 (11.1) | 4 (100) | 0 | 1 (25) | 5 (71.4) | 0 |
| Allo-SCT | 0 | 3 (75) | | 0 | 4 (80) | |
| Auto-SCT | 0 | 1 (25) | | 0 | 1 (20) | |
| Auto-SCT + CAR T | 1 | 0 | | 1 | 0 | |
| **Antibody response - number of patients with data available** | | | | | | |
| 28 days after the last immunizing event | 9 | 4 | 1 | 4 | 7 | 1 |
| **T-cell response - number of patients with data available** | | | | | | |
| 28 days after the last immunizing event | 3 | 3 | 0 | 4 | 5 | 0 |

[a] Patients with 2 immunizing events; either an infection followed by a vaccination or an infection after a vaccination or a vaccination followed by a vaccination and infection that occurred < 10 days of each other counting as 2 immunizing events.

[b] Patients with 3 immunizing events; being infected before, between or after 2-dose vaccination.

[c] Defined as receiving chemotherapy or immunotherapy less (Tx < 6 weeks) or more (Tx > 6 weeks) than 6 weeks before 1st immunizing event or having no history of chemotherapy or immunotherapy (No Tx)

[d] Non malignancies: patients with Fanconi Anemia and a history of HSCT

[e] IVIG (intravenous immunoglobulin) <2 months prior to 1st vaccination or SARS-CoV-2 infection.

[f] Number of days between HSCT and first vaccination varied between 95-1186 days.

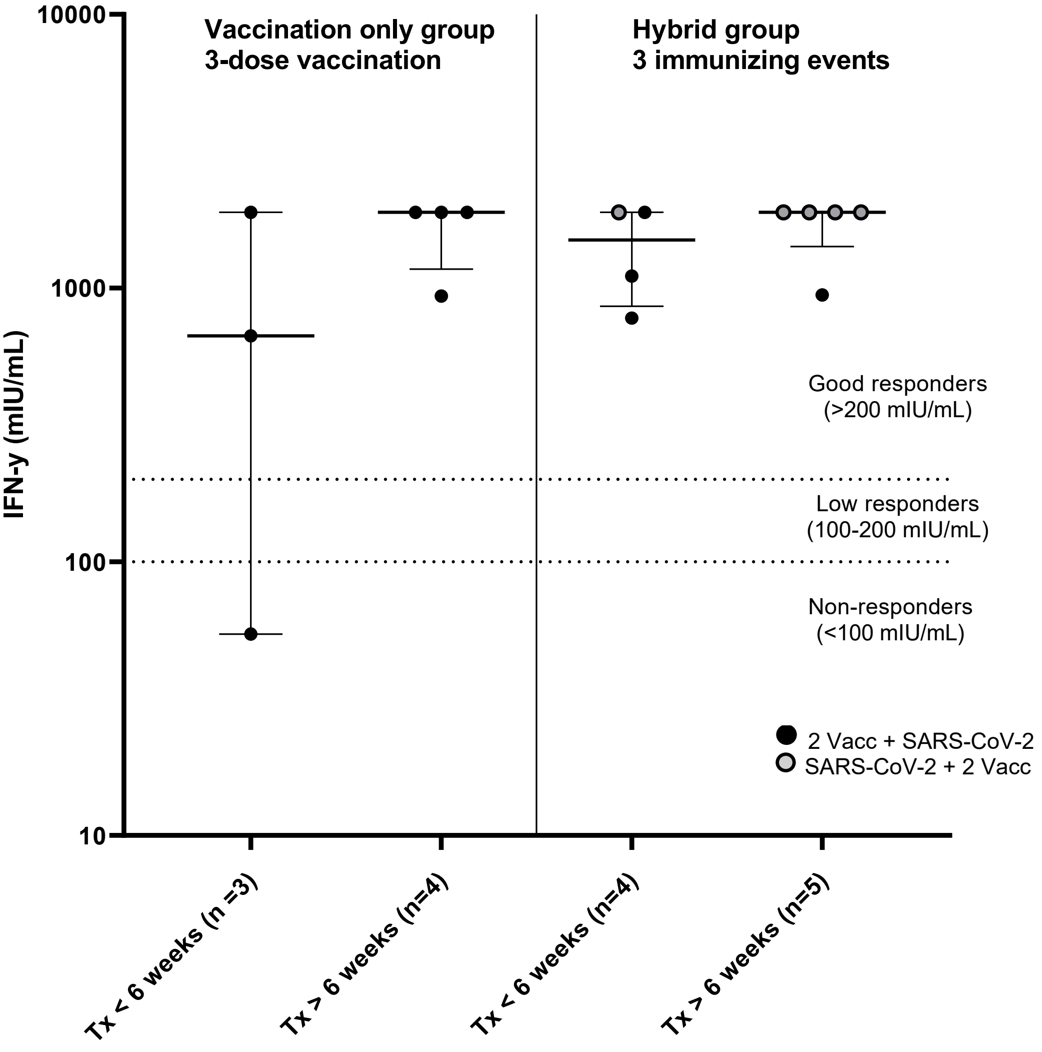


**Supplementary Figure S1. SARS-CoV-2 specific T-cell response following 3-dose vaccination or 3 immunizing events.** SARS-CoV-2 specific T-cell response at 4 weeks after 3-dose vaccination in patients aged 12-17 years or 3 immunizing events in patients aged 5-17 years. Patients were divided into two groups consisting of patients with immuno- or chemo-therapy < 6 weeks before 1st vaccination or 1st immunizing event (Tx < 6 weeks) or > 6 weeks before 1st vaccination or 1st immunizing event (Tx > 6 weeks). In the hybrid group patients were further categorized as having 2 vaccinations followed by SARS-CoV-2 infection (black circles) or being previously infected with SARS-CoV-2 followed by 2-dose vaccination (grey circles).
